# Affordability of Different Isocaloric Healthy Diets in Germany—An Assessment of Food Prices for Seven Distinct Food Patterns

**DOI:** 10.3390/nu13093037

**Published:** 2021-08-30

**Authors:** Stefan Kabisch, Sören Wenschuh, Palina Buccellato, Joachim Spranger, Andreas F.H. Pfeiffer

**Affiliations:** 1 Department of Endocrinology and Metabolic Medicine, Campus Benjamin Franklin, Charité University Medicine, Hindenburgdamm 30, 12203 Berlin, Germany; joachim.spranger@charite.de (J.S.); afhp@charite.de (A.F.H.P.); 2 Deutsches Zentrum für Diabetesforschung e.V., Geschäftsstelle am Helmholtz-Zentrum München, Ingolstädter Landstraße 1, 85764 Neuherberg, Germany; palina.zytner@gmx.net; 3 German Institute of Human Nutrition Potsdam-Rehbrücke, Department of Clinical Nutrition, Arthur-Scheunert-Allee 155, 14558 Nuthetal, Germany; 4 German Institute of Human Nutrition Potsdam-Rehbrücke, Department of Nutrition and Gerontology, Arthur-Scheunert-Allee 155, 14558 Nuthetal, Germany

**Keywords:** food pricing, low-carb, low-fat, vegan diet, vegetarian diet, Mediterranean diet, high-protein diet, affordability

## Abstract

Affordability of different isocaloric healthy diets in Germany—an assessment of food prices for seven distinct food patterns Background: For decades, low-fat diets were recommended as the ideal food pattern to prevent obesity, type 2 diabetes and their long-term complications. Nowadays, several alternatives considering sources and quantity of protein, fat and carbohydrates have arisen and clinical evidence supports all of them for at least some metabolic outcomes. Given this variety in diets and the lack of a single ideal diet, one must evaluate if patients at risk, many of which having a lower income, can actually afford these diets. Aim: We modelled four-week food plans for a typical family of two adults and two school children based on seven different dietary patterns: highly processed standard omnivore diet (HPSD), freshly cooked standard omnivore diet (FCSD), both with German average dietary composition, low-protein vegan diet (VeganD), low-fat vegetarian diet (VegetD), low-fat omnivore diet (LFD), Mediterranean diet (MedD) and high-fat moderate-carb diet (MCD). The isocaloric diets were designed with typical menu variation for all meal times. We then assessed the lowest possible prices for all necessary grocery items in 12 different supermarket chains, avoiding organic foods, special offers, advertised exotic super foods and luxury articles. Prices for dietary patterns were compared in total, stratified by meal time and by food groups. Results: Among all seven dietary patterns, price dispersion by supermarket chains was 12–16%. Lowest average costs were calculated for the VegetD and the FCSD, followed by HPSD, LFD, VeganD, MedD and—on top—MCD. VeganD, MedD and MCD were about 16%, 23% and 67% more expensive compared to the FCSD. Major food groups determining prices for all diets are vegetables, salads and animal-derived products. Calculations for social welfare severely underestimate expenses for any kind of diet. Conclusions: Food prices are a relevant factor for healthy food choices. Food purchasing is financially challenging for persons with very low income in Germany. Fresh-cooked plant-based diets are less pricy than the unhealthy HPSD. Diets with reduced carbohydrate content are considerably more expensive, limiting their use for people with low income. Minimum wage and financial support for long-term unemployed people in Germany are insufficient to assure a healthy lifestyle.

## 1. Introduction

Non-communicable diseases such as obesity, type 2 diabetes (T2DM), hypertension, dyslipidemia, gout and non-alcoholic fatty liver disease—all being part of or connected to the Metabolic Syndrome—contribute to severe long-term complications and premature death, causing suffering, disability and vast economic burdens to the health care system and the overall society throughout the world. A healthy diet can help to prevent all these disorders in the majority of people at risk, thus securing quality of life and saving money. However, despite various campaigns on health risks due to a poor diet, long-term compliance to any kind of healthier lifestyle is limited [1,2,3]. For the last decades, the low-fat diet received the strongest support from both industry and scientific advisors, but failed to be followed by most people. Currently, the debate on the ideal diet is covering several alternative options with possible superior effects on metabolic and long-term outcomes. Compared to low-fat diets, low-carb diets seem to be stronger in reducing glucose levels [4]. The Mediterranean pattern lowers the risk for stroke and myocardial infarction [5]. Isocaloric high-protein diets are very effective in improving the lipid profile and lowering liver fat content [6]. Vegan and vegetarian diets are superior with respect to inflammation and cholesterol levels [7,8]. All healthy diets share a higher content of fresh vegetables, non-processed foods and a reduced intake of free sugars. All of them seem to improve metabolic outcomes irrespective of weight loss and are therefore suitable as long-term food pattern for all kinds of subjects, almost independent of age, BMI and co-morbidity [4,5,6,7,8].

However, studies on these diets show that dietary compliance rapidly declines after short time [2]. Palatability, lack of variation, metabolic and gastrointestinal side effects are typical reasons for a loss of adherence in an investigational setting with financial reimbursements [9,10,11]. Outside of clinical trials, when people are bound to purchase all food items by themselves, health and food literacy, time constraints for meal preparation, but also affordability of a certain diet add up as additional factors for food choice and potential incompliance to diet recommendations [12].

Previous studies assessed the relation of certain food patterns and their costs for the individual customer in real-world settings from all over the globe. In the United States of America, daily expenses for the common diet (including food and beverages) were considerably lower compared to almost all recommended diets, except for the vegetarian diet. This survey also showed differences between ethnicities within the same country [13]. An interaction between ethnicity, the healthiness of a diet, and its costs was also seen in studies in cohorts from the Netherlands, Turkey, Morocco and Suriname, additionally highlighting a contribution of educational status. Higher education increased the price gap between healthy and unhealthy food, possibly indicating a partial influence of luxury products or unnecessary super foods [14]. Even in Mediterranean countries, the Western diet is considerably cheaper than the more healthy Mediterranean diet [15]. This also applies to children [16,17]. There is even some epidemiological evidence for a combined effect of food prices, food choice and metabolic outcome (obesity or glycemia) [18,19]. Higher costs due to a Mediterranean diet are corroborated by other surveys from the UK [20].

The low-carb diet, being another common alternative for low-fat, was also reported to require a roughly 19% larger family budget compared to standard diet in New Zealand [21].

A typical finding is also, that energy-dense food is less expensive than other products, indicating an effect of mostly highly-processed items [22]. However, the interaction between energy density, nutritional value and price is not unambiguous. Nuts and olive oil are considered healthy, despite their caloric content, and may be expensive. Low-energy beverages can be pricy or inexpensive [23].

Low prices for highly-processed foods are quite consistent when compared between different countries, while unprocessed foods (fruits, vegetables, but also meat) show a great variety in pricing even in countries of similar socioeconomic structure [24].

Higher or even rising prices for (healthy) food wouldn’t be a social problem, if wages and welfare would be sufficient to cover these expenses. However, there is evidence for some European regions, where food deprivation is partially linked to insufficient income. For Germany, this association was not found [25]. Also, German children investigated in the DOrtmund Nutritional and Anthropometric Longitudinally Designed (DONALD) study seem to be able to improve dietary quality without necessarily spending more money [26]. In adolescents of the same cohort, this balance deteriorates, but exchanging meat for fruits and vegetables appears to counteract a possible increase in dietary costs when introducing a healthier diet [27]. In other countries, e.g., Iran, healthy and unhealthy foods are priced similarly [28].

As there is only very limited data for Germany, we aimed for a systematic analysis of food prices for various dietary patterns which are considered to be either population standard or a healthy alternative. This was done for a hypothetical four-person household, for a duration of four weeks and covered twelve different supermarket and discounter chains in Berlin. As the current Coronavirus 2019 pandemic provides a realistic lockdown or distancing scenario with a combination of working from home and remote schooling, all dietary plans are designed to exclude take away, cafeterias, restaurants and other external options to eat meals.

## 2. Research Design and Methods

We modelled dietary plans for an average four-person household (one woman and one man between 21 and 51 years of age, one girl and one boy between 10 and 13 years of age) covering a period of four weeks. All meals are designed with sufficient variation for each type of dietary pattern: three variations of breakfast, seven alternative meals for lunch, three different meal options for dinner and five kinds of snacks. We aimed for a caloric proportion of 30% for each main meal and 10% for snacks. All diets are planned as isocaloric patterns covering the required energy intake based on a physical activity level of 1.6 for all four family members, mirroring previous similar assessments [21]. This led to an estimated energy intake of 2000 kcal, 2200 kcal, 2100 kcal and 2700 kcal for female and male children and adults, respectively. We modelled seven dietary patterns:

German average standard diet, resembling 43 energy% of carbohydrates, 37 energy % of fat and 17 energy% of protein, with 20 g of total fiber. This diet was designed either based on

(1) Highly processed standard omnivore diet (HPSD)
(2) Freshly cooked standard omnivore diet (FCSD)

Five alternative freshly cooked healthy eating patterns, being defined as

(1) Low-fat, low-protein vegan diet (VeganD)
(2) Low-fat, normal-protein vegetarian diet (VegetD)
(3) Low-fat normal-protein omnivore diet (LFD)
(4) Medium-fat, normal-protein Mediterranean omnivore diet (MedD)
(5) High-fat, moderate-carb omnivore diet (MCD)

Dietary targets and thresholds for all seven dietary patterns are described in detail in Table 1. Diets #2–7 were designed to abstain from highly-processed products. The vegan diet, however required some of these products in order to achieve protein recommendations. We avoided the implementation of expensive, unnecessary “super foods” and luxury articles and instead chose food products that would be available in regular supermarkets or discounters. For all healthy diets (#3–#7) we also aimed to fulfill the recommendations for micronutrients based on the guidelines by the German Nutrition Society (Deutsche Gesellschaft für Ernährung, DGE) [29]. We adapted the diets to achieve the goals for fiber, sugar, salt, uric acid, cholesterol, calcium, magnesium, iron, Vitamin C, E and B_12_. For all healthy diets, we also assured the “five-a-day rule”, assuring intake of vegetables and fruits five times a day. Other DGE rules such as the recommended n6/n3-ratio of PUFAs (5:1) and limitation of meat intake were not covered, as most healthy diets would clearly violate these goals anyway. Beverages included 2.15 L for children and 2.6 L for adults by bottled mineral water, only. For HPSD, fruit juices and Café Crema were added as typical highly-processed high-energy drinks. Alcoholic beverages were not included into our meal plans.

The respective four-week food plans were composed and analysed using PRODI 6.2, which includes the Bundeslebensmittelschlüssel and the MONICA food list [30]. Dietary compositions of all food plans are presented in Table 2 (Macronutrients) and Table 3 (Micronutrients).

For all articles the lowest possible regular price for a standard product (no special offers, no seasonal offers for processed foods) was determined in twelve different supermarkets and discounters, belonging to the companies ALDI Nord, Edeka, Kaufland, LIDL, Netto, Netto plus, Norma, Penny, Spar, Real, REWE and Metro. All grocery stores are located in Berlin, covering an urban infrastructure with predominantly average-to-low income households. The assessment was done in January and February 2021.

We excluded special prices, seasonal discounts, but otherwise chose the cheapest product for the respective item, irrespective of label, NutriScore or any other aspect. Therefore, “organic” foods, meat products with specific ethical consideration (animal welfare) and any other special branches of food production were not part of our grocery list. If possible or necessary, frozen fruits and vegetables were chosen over fresh ones in order to reduce the theoretical expenses. In total, prices for 134 items were collected. These items were categorized according to ten food groups: (1) starchy plant products, (2) vegetables and salads, (3) fruits, (4) milk and dairy products, (5) eggs, fish, processed and unprocessed meat products, (6) oils and fats, (7) beverages, (8) spices and sauces, (9) highly processed food items and ready-to-eat meals, and (10) snacks and sweets (Supplementary materials). Highly processed food items covered main dish products for which natural ingredients underwent extensive grinding, extraction of fiber or minerals and heating, and which contain a relevant amount of added fat, sugar, salt and other dietary additives. This covers for example white bread or breakfast cereals, but also fruit joghurt, tofu, tempeh and seitan.

## 3. Statistical Analyses

Food prices between dietary patterns were compared using unpaired *t*-tests (given normal distribution of the data). This assessment was done for the entire four-week intake pattern, but also separately for breakfast, lunch and dinner. All data are presented as means, calculated from the data of the twelve respective supermarkets and discounters. The results were considered significantly different if *p* < 0.05. All statistical analyses were performed using SPSS for Windows program version 25.0 (SPSS Inc., Chicago, IL, USA).

## 4. Results

For a four-person household, prices for four weeks of food and beverages summed up to average costs of 652 € to 1121 €, depending on the selected diet. Under additional consideration of individual supermarkets, the prices ranged from 498 € to 1322 €. Price dispersion between supermarkets ranged between 12 and 16% of the mean prices for all diets. Prices were lowest for the VegetD and the FCSD, followed by the HPSD and the LFD, the VeganD, the MedD—on top of the list—the MCD. The MCD was significantly more expensive than any other diet (*p* < 0.001 for all comparisons), the MedD was significantly pricier than HPSD, FCSD, VegetD and LFD (*p* < 0.01 for all comparisons). The VegetD was significantly less pricy than VeganD, MedD and MCD (*p* < 0.01, *p* < 0.001, *p* < 0.001) (Table 4).

Even though all diets were designed to attribute each 30% of energy to each main meal and 10% to snacks, price distribution for these four meal times were disproportionally skewed. In all diets, snacks accounted for more than 10% of the budget, while almost all main meals had a lower price-to-energy ratio (Table 5).

We also investigated, to which percentage each food group contributed to the overall price of the diets. We used the DGE categorization of food groups for this purpose [31]. Main drivers for higher prices, as seen in the food groups, are vegetables and salads for all healthy diets, milk and dairy for the VegetD, eggs, fish and meat for standard diets and MCD (Table 6).

## 5. Discussion

To our knowledge, our assessment is the first comprehensive analysis of food prices for a broad selection of fictional healthy and unhealthy diets in Germany. We demonstrate, that most healthy diets are more expensive than the German average diet, be it highly processed or freshly cooked. The use of highly-processed ready-to-eat meals does not seem to save money, as a freshly cooked meal of the same composition appears to be 5% cheaper. The only diet which is both healthy and less expensive is the vegetarian diet, requiring about 650 € for a regular four-person family within four weeks. The low-fat diet does not save money in comparison to standard diet. The vegan diet, the Mediterranean diet and most impressively the moderate-carb diet cost 16%, 23% and 67% more than the FCSD. Despite covering only a minor portion of caloric intake, snack time overproportionally contributes to the grocery budget. For all freshly cooked healthy diets, vegetables, salads and meat products (if present) bear the highest financial load.

Our paper confirms previous assessments on food prices from the USA, Spain and the UK [13,15,16,17,20]. In all these publications, the Western diet turned out to be cheaper than healthier alternatives, in particular the Mediterranean diet. We can pinpoint this difference to higher expenses for vegetables, salads, spices and sauces.

As many other healthy diets are also plant-based (vegan and vegetarian diet), similar effects would be expected. However, we only notice a higher budgetary demand for the vegan, but not the vegetarian diet. Money seems to be saved when avoiding meat products, while milk and dairy (most prominent in our vegetarian diet), do not appear to contribute to a higher financial burden. The vegan diet exceeds the costs for a vegetarian and the standard diets, which can be explained by the need for rather specific plant-based foods which provide not only carbohydrates, but also fat and protein. Achieving dietary recommendations when undergoing a vegan diet is almost impossible if highly processed protein-rich plant foods (tofu, seitan…) are not consumed. Additional expenses for supplements (e.g., vitamin B_12_ and D) need to be considered [32,33,34]

Isolated carbohydrates are apparently the cheapest nutrients. Diets with carbohydrate restriction—including the Mediterranean diet—may be very effective for metabolic amelioration, but require a substantially higher household budget for food. Moderate- and even more low-carb diets are predominantly composed of leafy and other low-energy vegetables and all kinds of animal-based foods. This combination is crucial for the excess in food pricing, supported by data from the USA [13].

A moderate-carb diet can be conducted isocaloric, making the financial comparison to other isocaloric diets plausible. On the other hand, low-carb, even ketogenic diets are most often consumed in order to lose weight, thus requiring fewer products. Being hypocaloric and highly filling, their *relative* excess in monetary demands might be partially compensated by actually lower *absolute* amounts of food.

There is also a moderate potential to save money by consuming tap water instead of bottled mineral water. In our assessment, even this inexpensive discounter beverage accounted for 6–11% of the dietary expenses.

We are surprised to find, that none of our diets, including German standard diets and even the cheapest alternative (VegetD), can be afforded with a monthly budget of 150 € per adult. In Germany, this particular sum is the amount of money, which the most basic social welfare program (“ALG II”) attributes to expenses for food and beverages. It is clear, that unemployed people and those with very low income, requiring this financial support, need to cut other elementary expenses in order to afford any kind of (healthy or unhealthy) diet. Even single and family household living on minimal wage (currently 9.60 € per hour in Germany) are hardly capable to afford a substantially healthy diet. This finding is even more important, as metabolic disorders are connected to socioeconomic status. Obesity, the Metabolic Syndrome and type 2 diabetes are more common among low-income households [35]. Especially these households require professional dietary consultation to assure health and food literacy, but also sufficient financial support in order to effectively change their unhealthy dietary pattern. Buying “organic” foods, animal-based products which assure animal welfare and specific healthy items (such as linola oil, nuts, berries) will considerable increase the expenses on top of our estimations. Also, these households might suffer from time constraints, leading to the consumption of ready-to-eat meals which do not require a long preparation time. All freshly cooked diets in our assessment include three daily meals, for which several ingredients need to be mixed or heated. Each of these diets require some sort of critical consideration of cooking techniques in order to assure a pleasant food texture, taste and appearance, while maintaining the dietary goals. All diets contain a set of raw components, which are left uncooked, and others, that have to be grilled, baked, boiled or steamed. We do not expect any diet to be especially difficult to prepare.

Our analysis has several strengths. It is the first head-to-head comparison of several diets, including a Western-type average diet with or without highly-processed foods and a variety of healthy alternatives. We conceptualized isocaloric diets, fulfilling as many dietary recommendations as possible. Nevertheless, we prioritized low prices, leading to a very conservative calculation. We excluded luxury foods, super foods, “organic” products, all of which are neither necessary for a healthy diet nor able to save money. On the other hand, we excluded special offers and seasonal sales in order to provide comparable data throughout the year. We assured sufficient variation in the individual dietary patterns by generating several options for each meal time. Last, we extracted food prices from their original source in actual supermarkets, located in an urban environment with lower socioeconomic status.

Of course we are aware of several limitations of our work. Food prices are affected by regionality, even within a country, a province or a large city. By choosing a low-income urban area to assess food prices, our calculation approximates the potential absolute minimum of food prices in Berlin and Germany. However, extrapolations to other regions and countries are difficult. Our “menu from the scratch” may not necessarily reflect, what people actually eat. Unhealthy and healthy dietary patterns are highly diverse, often with overlapping food groups from both sides. Contrasting two standard diets with the same dishes based on processed and unprocessed food, only, showed the small but existing potential to reduce food costs by home-cooking. Most dishes of the German cuisine are available as ready-to-eat meals, which might help to save preparation time, but apparently not to save money. Surely, these savings do not account for expenses in preparation time and electricity or gas. There may be also seasonal effects, especially when it comes to plant-based diets. Not all vegetables and fruits are available throughout the year, and if, for the same price. Optional selection of frozen foods for our menu plans was an opportunity to outrule seasonal effects.

In summary, our analysis provides an exemplary overview of the food pricing landscape in Germany for unhealthy and healthy dietary patterns. People can marginally save money by deciding to cook by themselves or switching to a vegetarian diet. However, the most recommended diets (from a metabolic point of view)—Mediterranean diet and other carbohydrate-reduced schemes—require 20–60% more household budget for food. Despite having relatively low food prices in Germany (in comparison to other European countries), standard diets and especially healthy diets are hardly affordable for low-income households. Dietary recommendations need to take into account limitations to compliance to due financial issues and politics should re-assess social welfare programs and minimum wage in accordance to our findings.

## Figures and Tables

**Table 1:** Dietary targets for the model diets.

Diet	Dietary Goals
HPSD	40–45% carbohydrates, 15–20% saturated fat, 15–20% unsaturated fat, 15–20% protein
FCSD	40–45% carbohydrates, 15–20% saturated fat, 15–20% unsaturated fat, 15–20% protein
VeganD	50–60% carbohydrates, <10% saturated fat, <20% unsaturated fat, <15% protein
VegetD	50–60% carbohydrates, <15% saturated fat, <15% unsaturated fat, 15–20% protein
LFD	50–60% carbohydrates, <15% saturated fat, <15% unsaturated fat, 15–20% protein
MedD	40–45% carbohydrates, <15% saturated fat, 20–25% unsaturated fat, 15–20% protein
MCD	25–30% carbohydrates, 25–30% saturated fat, 20–25% unsaturated fat, 20% protein

FCSD: Freshly cooked standard omnivore diet, HPSD: Highly processed standard omnivore diet, LFD: Low-fat normal-protein omnivore diet, MCD: High-fat, moderate-carb omnivore diet, MedD: Medium-fat, normal-protein Mediterranean omnivore diet, VeganD: Low-fat, low-protein vegan diet, VegetD: Low-fat, normal-protein vegetarian diet.

**Table 2:** Actual dietary macronutrient composition of the model diets.

Diet	Carbohydrates	Fibre	Saturated Fat	Unsaturated Fat	Protein
HPSD	45%	1%	17%	17%	20%
FCSD	46%	3%	16%	17%	19%
VeganD	56%	5%	5%	22%	13%
VegetD	54%	4%	16%	11%	17%
LFD	56%	5%	7%	13%	20%
MedD	45%	3%	16%	21%	16%
MCD	30%	3%	27%	21%	20%

All data are means. FCSD: Freshly cooked standard omnivore diet, HPSD: Highly processed standard omnivore diet, LFD: Low-fat normal-protein omnivore diet, MCD: High-fat, moderate-carb omnivore diet, MedD: Medium-fat, normal-protein Mediterranean omnivore diet, VeganD: Low-fat, low-protein vegan diet, VegetD: Low-fat, normal-protein vegetarian diet.

**Table 3:** Micronutrient composition of the model diets, based on 2000 kcal per day.

Diet	Fiber (g/d)	Sugar (g/d)	Salt (g/d)	Uric Acid (mg/d)	Cholesterol (mg/d)	Calcium (mg/d)	Magnesium (mg/d)	Iron (mg/d)	Vit. C (mg/d)	Vit. E (mg/d)	Vit. B_12_ (mg/d)
HPSD	12.5	51	4.1	233	169	675	177	4.1	101	8.6	3.0
FCSD	26.4	54	6.3	353	150	750	361	12.0	106	7.3	3.3
VeganD	47.4	56	3.7	407	1	632	523	16.9	311	18.2	0.2
VegetD	37.3	48	2.6	255	182	1079	409	13.7	232	8.8	3.2
LFD	46.5	52	6.4	374	49	835	466	15.6	283	13.7	2.4
MedD	34.0	45	6.7	238	244	983	432	14.3	256	15.4	3.4
MCD	27.9	31	4.5	370	809	1209	356	13.6	426	17.0	9.1

All data are means. FCSD: Freshly cooked standard omnivore diet, HPSD: Highly processed standard omnivore diet, LFD: Low-fat normal-protein omnivore diet, MCD: High-fat, moderate-carb omnivore diet, MedD: Medium-fat, normal-protein Mediterranean omnivore diet, VeganD: Low-fat, low-protein vegan diet, VegetD: Low-fat, normal-protein vegetarian diet.

**Table 4:** Average grocery prices by diet.

	HPSD	FCSD	VeganD	VegetD	LFD	MedD	MCD
Mean prices (four persons)	701.03 €	670.02 €	777.98 €	651.63 €	711.50 €	824.21 €	1120.75 €
Mean prices (female child)	155.78 €	148.89 €	172.88 €	144.81 €	158.11 €	183.16 €	249.06 €
Mean prices (male child)	171.36 €	163.78 €	190.17 €	159.29 €	173.92 €	201.47 €	273.96 €
Mean prices (female adult)	163.57 €	156.34 €	181.53 €	152.05 €	166.02 €	192.32 €	261.51 €
Mean prices (male adult)	210.31 €	201.01 €	233.39 €	195.49 €	213.45 €	247.26 €	336.23 €

All data are means. FCSD: Freshly cooked standard omnivore diet, HPSD: Highly processed standard omnivore diet, LFD: Low-fat normal-protein omnivore diet, MCD: High-fat, moderate-carb omnivore diet, MedD: Medium-fat, normal-protein Mediterranean omnivore diet, VeganD: Low-fat, low-protein vegan diet, VegetD: Low-fat, normal-protein vegetarian diet.

**Table 5:** Percentage of food prices according to meal time.

	HPSD	FCSD	VeganD	VegetD	LFD	MedD	MCD
Price fraction/breakfast	27%	27%	30%	25%	23%	22%	32%
Price fraction/lunch	30%	29%	27%	27%	23%	24%	28%
Price fraction/dinner	27%	28%	30%	26%	42%	34%	26%
Price fraction/snack	16%	16%	13%	22%	12%	21%	14%

All data are means. FCSD: Freshly cooked standard omnivore diet, HPSD: Highly processed standard omnivore diet, LFD: Low-fat normal-protein omnivore diet, MCD: High-fat, moderate-carb omnivore diet, MedD: Medium-fat, normal-protein Mediterranean omnivore diet, VeganD: Low-fat, low-protein vegan diet, VegetD: Low-fat, normal-protein vegetarian diet.

**Table 6:** Price proportions according to food groups.

	HPSD	FCSD	VeganD	VegetD	LFD	MedD	MCD
Starchy plant products	4%	13%	20%	19%	19%	9%	2%
Vegetables and salads	5%	13%	29%	24%	32%	30%	40%
Fruits	7%	12%	21%	16%	22%	11%	10%
Milk and dairy	16%	12%	0%	20%	8%	15%	14%
Eggs, fish and meat	29%	33%	0%	2%	7%	14%	24%
Oil and fats	0%	2%	2%	1%	1%	2%	2%
Beverages	12%	9%	11%	10%	9%	8%	6%
Spices and sauces	1%	2%	8%	7%	2%	10%	3%
Highly processed products	24%	0%	7%	0%	0%	0%	0%
Snacks and sweets	1%	3%	3%	1%	1%	2%	0%

All data are means. FCSD: Freshly cooked standard omnivore diet, HPSD: Highly processed standard omnivore diet, LFD: Low-fat normal-protein omnivore diet, MCD: High-fat, moderate-carb omnivore diet, MedD: Medium-fat, normal-protein Mediterranean omnivore diet, VeganD: Low-fat, low-protein vegan diet, VegetD: Low-fat, normal-protein vegetarian diet.

## Data Availability

Data sets are available by request to the corresponding author.

## Supplementary Materials

The following are available online at https://www.mdpi.com/article/10.3390/nu13093037/s1, Supplementary materials: List of food and beverage items.

## Abbreviations

DGE	German Nutrition Society (Deutsche Gesellschaft für Ernährung)
DONALD	DOrtmund Nutritional and Anthropometric Longitudinally Designed
FCSD	Freshly cooked standard omnivore diet
HPSD	Highly processed standard omnivore diet
LFD	Low-fat normal-protein omnivore diet
MCD	High-fatmoderate-carb omnivore diet
MedD	Medium-fatnormal-protein Mediterranean omnivore diet
T2DM	type 2 diabetes mellitus
VeganD	Low-fatlow-protein vegan diet
VegetD	Low-fatnormal-protein vegetarian diet
