# Supplementary material for: Affordability of Different Isocaloric Healthy Diets in Germany—An Assessment of Food Prices for Seven Distinct Food Patterns
Source: Nutrients. 2021 Aug 30;13(9):3037. doi: 10.3390/nu13093037 (PMC8472114; doi:10.3390/nu13093037)
Supplement: Supplementary file 1 [file nutrients-13-03037-s001.zip › nutrients-1312264-supplementary.pdf]

nutrients MDPI

Affordability of Different Isocaloric Healthy Diets in Germany—An Assessment of Food Prices for Seven Distinct Food Patterns

Supplementary materials: List of food and beverage items

Food group 1: Starchy plant products

Whole wheat toast	Bread roll	Soft oat meal flakes	Tortilla (wheat)	Plain corn flakes
Butter toast	Grain rolls	Pasta (durum wheat)	Couscous	Dumpling dough
White wheat crispbread	English muffin	Pasta sheets (durum wheat)	Bulgur	Pizza dough
Whole rye crispbread	Baguette	Potatoes	Rice	Breadcrumbs
Whole grain bread	Oatmeal	Gnocchi		

Food group 2: Vegetables and salads

Paprika (red)	Basil	Spinach (frozen)	Capers (can)	Kohlrabi
Broccoli	Garlic	Green beans (frozen)	Cucumber	Lentils (raw)
Peas (frozen)	Parsley	Ruccola	Corn (can)	Red cabbage (frozen)
Carrots	Celery	Cauliflower	Leaf lettuce	
Onion	Tomatoes	Chickpeas	Beans (white)	
Zucchini	Olives (black)	Mushrooms	Kidney beans	

Food group 3: Fruits

Raspberries (frozen)	Tangerine	Grapes	Nut mix	Hazelnuts
Strawberries (frozen)	Pear	Lemon	Walnuts	
Pineapple	Apple	Banana	Pistachio	
Kiwi	Dates	Almonds	Linseed	

Food group 4: Milk and dairy products

Yoghurt with crispy grains	Cream cheese	Cream	Sheep cheese	Harzer cheese (hard cheese)
Gouda (slices)	Mozzarella (45 % fat)	Whipped cream	Camembert	Feta cheese
Rice pudding (chocolate)	Mozzarella (20 % fat)	Parmesan cheese	Yogurt (vanilla)	Grained cream cheese
Low fat creme cheese	Natural yogurt (normal fat)	Milk (1.5% fat)	Pudding (chocolate)	Emmentaler
Creme fraiche cheese	Natural yogurt (low fat)	Milk (3.5% fat)	Grilled / pan cheese	

Food group 5: Eggs, fish, processed and unprocessed meat products

Beef (meat)	Turkey breast (meat)	Salami sausage	Tuna (can)	Fish fingers (frozen)

nutrients

MDPI

Minced meat (pork / beef)	Schnitzel Cordon Bleu (frozen)	Cooked ham	Salmon (frozen)	Gourmet fillet
Chicken breast (meat)	Cracker sausage	Bacon	Sea bream / sea salmon fillet (frozen)	Chicken egg
Duck (frozen)	Turkey breast (sausage)	Wiener sausages	Fried fish (frozen)	

Food group 6: Oils and fats

Olive oil	Sunflower oil	Vinegar	Balsamic vinegar	Butter (salted)
Rapeseed oil	Corn oil			

Food group 7: Beverages

Mineral water	Apple spritzer	Fruit juice	Coconut milk	Rice drink
Café Crema	Orange juice			

Food group 8: Spices

Mustard	Seasoned Salt	Tomato paste	Vegetable broth	Oregano
Curry powder	Tomato sauce	Meatsoup	Pesto (red)	Thyme

Food group 9: Highly processed food items and ready-to-eat meals

Lasagna (frozen)	Pasta salad	Noodle soup	Dumplings (frozen)	Soy yogurt
Paella / rice pan (frozen)	Meat salad	Goulash (can)	Potato fries (frozen)	Tofu
Chili con carne (frozen)	Sandwich (with poultry / salami)	Pizza Margherita	Hummus	

Food group 10: Snacks and sweets

Chips (paprika)	Chocolate cream (spread)	Honey	Jam (orange)	Pretzel Sticks
Chocolate	Chocolate biscuit	Sugar	Jam (strawberry)	Gummy bears
